# Design, development and evaluation of registry software for upper limb disabilities

**DOI:** 10.1049/htl2.12115

**Published:** 2024-12-12

**Authors:** Khadijeh Moulaei, Abbas Sheikhtaheri, AliAkbar Haghdoost, Mansour Shahabi Nezhad, Kambiz Bahaadinbeigy

**Affiliations:** ^1^ Health Management and Economics Research Center Health Management Research Institute Iran University of Medical Sciences Tehran Iran; ^2^ Artificial Intelligence in Medical Sciences Research Center Smart University of Medical Sciences Tehran Iran; ^3^ Department of Health Information Management School of Health Management and Information Sciences Iran University of Medical Sciences Tehran Iran; ^4^ HIV/STI Surveillance Research Center and WHO Collaborating Center for HIV Surveillance Institute for Futures Studies in Health Kerman University of Medical Sciences Kerman Iran; ^5^ Department of Physical Therapy Faculty of Allied Medicine Kerman University of Medical Sciences Kerman Iran; ^6^ Clinical Workshops instructor Digital Health Specialist The Australian College of Rural and Remote Medicine (ACRRM) Brisbane Australia

**Keywords:** medical information systems, public domain software

## Abstract

Upper limb disabilities, if not managed, controlled and treated, significantly affect the physical and mental condition, daily activities and quality of life. Registries can help control and manage and even treat these disabilities by collecting clinical‐management data of upper limb disabilities. Therefore, the aim of this study is to design, develop and evaluate a registry system for upper limb disabilities in terms of usability. By having identified data elements in the exploratory phase, we developed our registry software using hypertext preprocessor (PHP) programming language in XAMPP software, version 8.1.10. The content and interface validity of the pre‐final version were assessed by 13 experts in the field of medical informatics and health information management. The registry has capabilities to create user profiles, record patient history, clinical records, independence in daily activities, mental health, and treatment processes. It can also generate statistical reports. Participants evaluated the registry's usability as “good” across different dimensions. The registry can help understand upper limb disabilities, improve care, reduce costs and errors, determine incidence and prevalence, evaluate prevention and treatment, and support research and policymaking. The registry can serve as a model for designing registries for other body disabilities.

## Graphical abstract

Upper limb disabilities can have a significant impact on an individual's physical and mental well‐being and daily life. The introduction of a registry system specifically tailored to upper limb disabilities is studied. The comprehensive range of data collected, including patient history, clinical records, mental health, and treatment processes, is unique to this registry. The ability to produce various statistical reports in different formats is a valuable feature for researchers and healthcare providers.

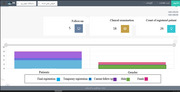

## 1 INTRODUCTION

Musculoskeletal disabilities and disorders create complex societal problems, often limiting a person's ability to meet daily needs like driving, using public transportation, eating, and obtaining health benefits from regular exercise [1]. These disorders are among the most common chronic diseases, significantly affecting physical activity, mental health, and quality of life, and they become more prevalent with age [2]. Upper limb disabilities are one type of musculoskeletal disorder [3].Upper limb disabilities include impaired movement and coordination of the arms, hands, and fingers, and usually limit a person's ability to perform activities such as eating, dressing, and washing [3]. Moreover, defects in the upper limbs often cause problems such as limitations in the range of movements or the sense and strength of the limbs, which in turn can lead to the inability of a person to function in life [4, 5]. Kalron et al. [6], note that upper limb dysfunction can lead to secondary diseases, reduced social, recreational, and occupational participation, and lower quality of life. The impact of these disabilities is more related to mental and psychosocial aspects than to physical measures [7]. Other studies [8, 9] have shown a significant correlation between upper limb disabilities and depression and anxiety. The sudden loss of motor function can profoundly affect a person's life, creating lasting physical and emotional burdens. Therefore, managing these disabilities is essential [10]. Therefore, one should look for a way to control and manage these disabilities.

Disease registries are crucial for managing and monitoring diseases and disabilities, providing key epidemiological information to deliver extensive patient services [11, 12]. Establishing a registry is vital for any country's healthcare management, involving continuous and systematic data collection on disease occurrence and related outcomes [13, 14]. These registries organize clinical and demographic patient information, support clinical research, monitor disease prevalence and incidence, and evaluate various aspects of healthcare processes and outcomes [14, 15]. This powerful tool can track disease progression, assess treatment changes and outcomes, evaluate prognosis and quality of life factors, and describe care patterns, including appropriateness and inequities in medical services [16]. It also identifies disease risk factors, reveals treatment differences between countries, saves money and time, improves data quality, fosters international research collaboration, and evaluates long‐term therapeutic interventions [17]. By organizing clinical and demographic patient information and supporting clinical research [15], these tools monitor disease prevalence and incidence, evaluate healthcare processes and outcomes [15], these tools monitor disease prevalence and incidence, evaluate healthcare processes and outcomes [14], and address research, care, and policy challenges [18]. Bae et al. [19], demonstrated that registries of upper limb congenital disabilities aid in research on societal impacts. Another study [20], by Bae et al. highlighted that continuous registration and longitudinal follow‐ups enhance understanding of upper limb function and psychosocial health in children.

To our knowledge, no study has been published to design, develop, and evaluate an upper limb disability registry. Only some studies, such as the two studies by Bae et al., have used data recorded in pre‐designed registries to identify and categorize congenital defects of upper limb disabilities [19] and to identify various functional effects of congenital hand differences on patients [20]. Therefore, the aim of the present study was to design, develop, and evaluate a registry for upper limb disabilities.

## 2 METHODS

In the first step, in order to determine the data elements necessary for the design of the minimum data set related to the design of the registry, a literature review was conducted on November 11, 2020, in PubMed, Web of Science, and Scopus databases. Management and clinical data necessary for registry design were extracted from the included studies. Then, based on the data set identified in the previous step, a questionnaire was designed. In the next step, the two‐round Delphi method was used for the final confirmation of the minimum data set required for the design of the registry. Twenty orthopedic, physical medicine and rehabilitation physicians and physiotherapists participated in the first and second round of Delphi and completed the questionnaire. This process and detailed information have already been published in another article [21].

Finally, 77 data items were finally confirmed by experts as essential data elements for the design of the minimum registry data set of upper limb disabilities.

According to the administrative and clinical data elements identified in the previous step, the prototype registry software for upper limb disabilities was designed and implemented using hypertext preprocessor (PHP) programming language in XAMPP software (XAMPP 8.1.10). Moreover, the database of this registry was designed using MySQL (MySQL Community Server 8.0.32). Then, in order to register and store the data of patients with upper limb disabilities, the software was launched at Kerman University of Medical Sciences.

In order to evaluate the usability of the software, the phone numbers of 18 experts in medical informatics and health information management were provided to us by the director of the Medical Informatics Association of Iran. An invitation letter was sent to these people through WhatsApp and email to participate in the study. Fifteen people accepted our invitation. Finally, according to the inclusion criteria, 13 people were included in the study. Two people were excluded from the study due to unwillingness to cooperate.

Inclusion criteria:

‐ History of registry design, implementation and evaluation
‐ History of research work in the field of registries
‐ Experience working with registries in medical centers and hospitals

Moreover, the exclusion criterion included the person's unwillingness to cooperate in the evaluation process.

The participants were asked to use this software for 1 month. Then, the fifth version of the standard questionnaire for user interface satisfaction (QUIS) was used to evaluate the usability of the software. The reliability of this questionnaire was confirmed with Cronbach's alpha of 0.92. This standard questionnaire has a 10‐point Likert scale and consists of six parts. These sections are respectively: addressing the identity of the participants (three questions), overall reaction to the software (six questions), screen (four questions), terminology and information used in the software (six questions), learning (six questions), software capabilities (five questions) [22]. We also added an open question titled “other usability problems” at the end of the questionnaire to identify other usability problems.

In the next step, the questionnaire was designed electronically through Google Form. Its link was sent to the participants via WhatsApp, Telegram, and email. Questionnaire data were analyzed using SPSS 23. The mean scores of 0–3 was classified as poor, 3. 1–6 as intermediate, and 6. 1–9 as good. It should be noted that the features of the software that got a poor score, those parts were modified.

## 3 RESULTS

**FIGURE 1:**
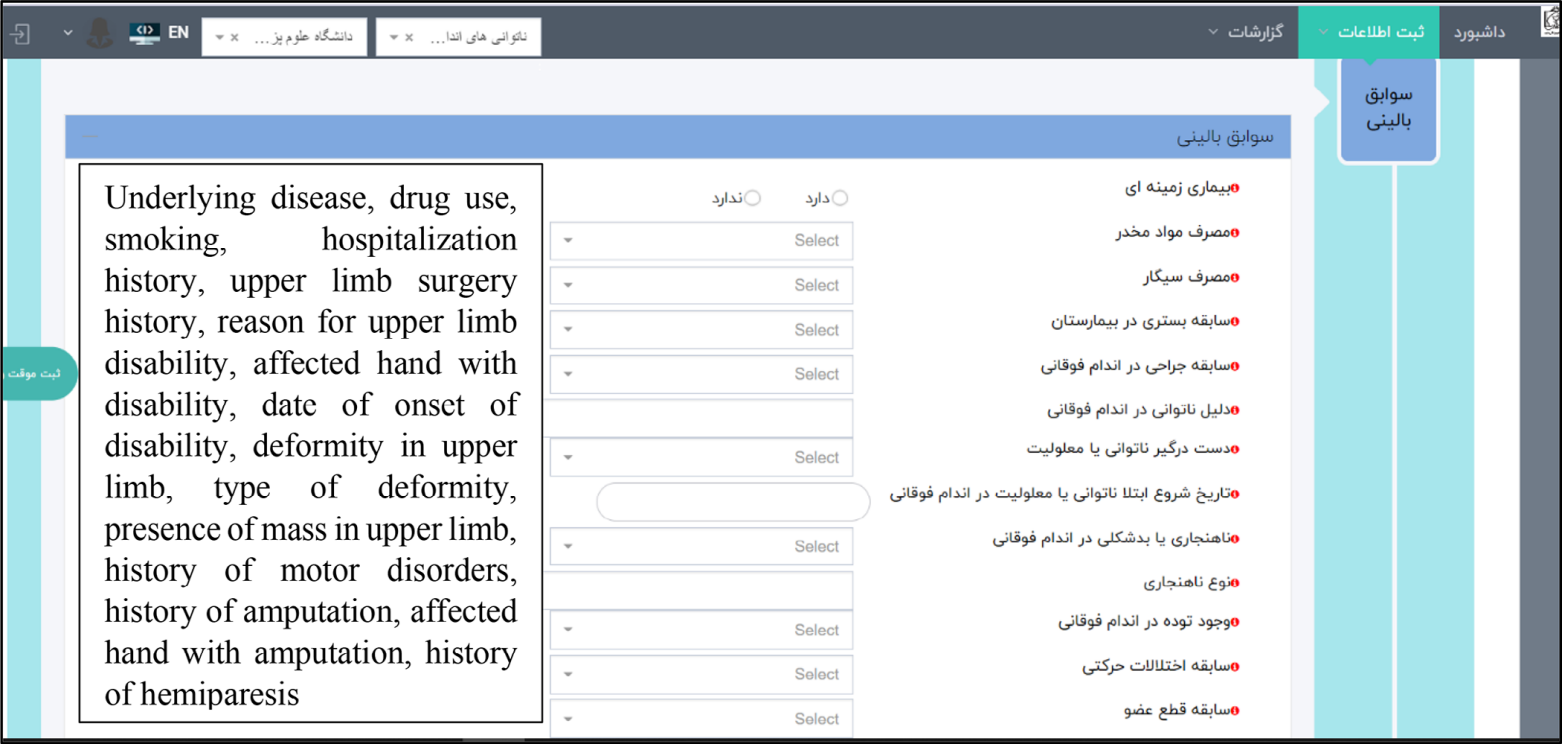
Past medical history. *Note: The English translations of the fields are listed in order on the left side of the figure.

**FIGURE 2:**
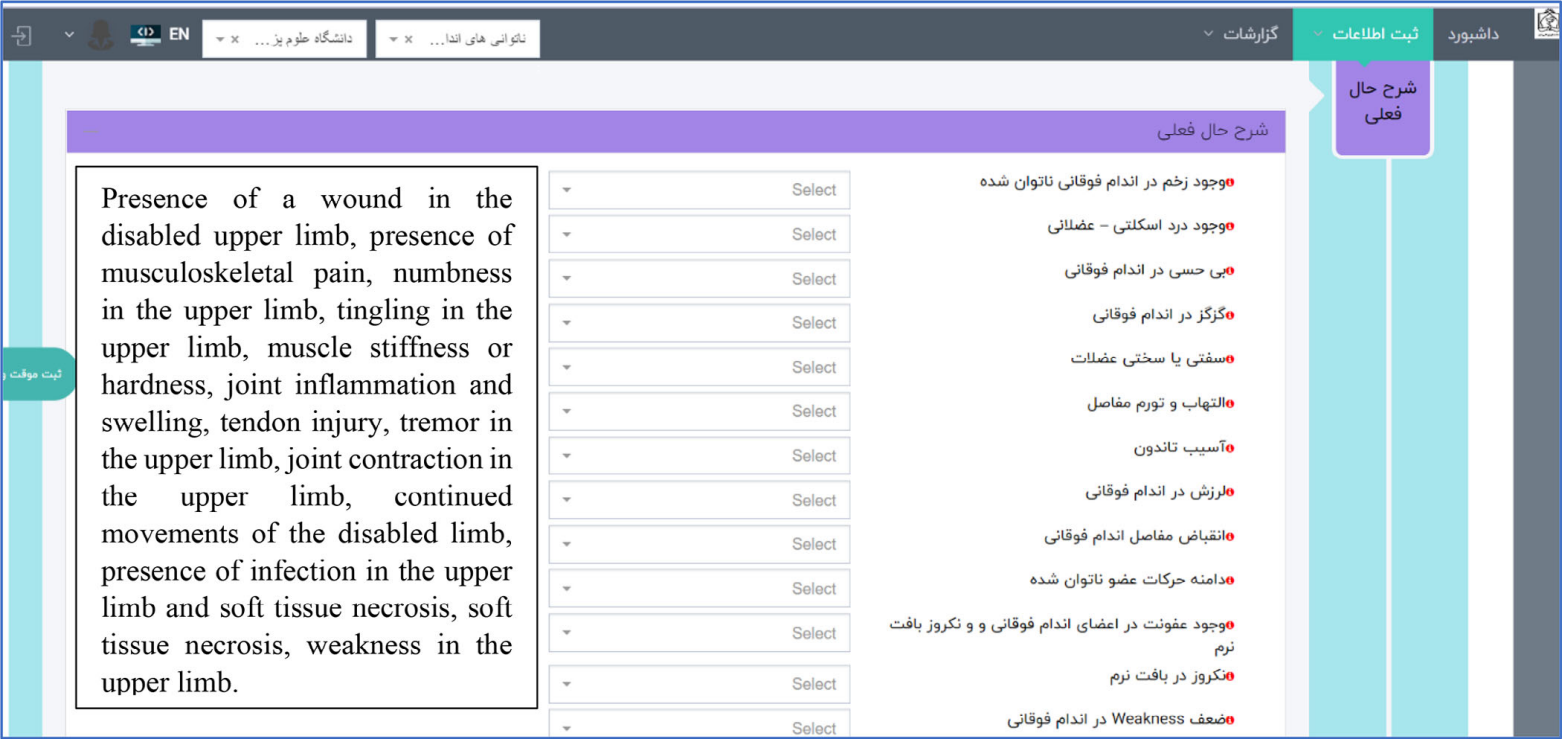
Current medical history of the patient. *Note: The English translations of the fields are listed in order on the left side of the figure.

**FIGURE 3:**
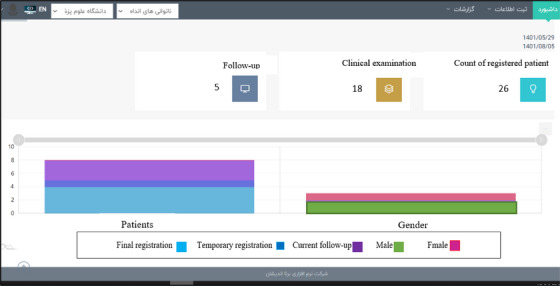
Dashboard.

After the final identification and approval of the data elements, the prototype of the software was designed and can be accessed through the link https://mdreg.mums.ac.ir/. This software has capabilities to create a profile for registry user, defining a new patient, and recording information such as the past medical history (Figure 1), the current medical history of the patient (Figure 2), the levels of individual independence of the patient in performing daily activities, information related to the patient's mental health problems, and the treatment processes performed for the patient. Also, this software has different menus including dashboard menu (Figure 3), information registration, reports, follow‐ups list, and patient search. Table 1 shows the capabilities and menus of the software.

**TABLE 1:** Capabilities and menus of the registry of upper limb disabilities.

Row	Capabilities and menus	Features and characteristics
1.	**Creating profiles for users to register information in software**	Defining authorized users to register information, defining a username and password for each user, logging in by entering the username and password, registering, editing and deleting the information of patients with upper limb disability.
2.	**Defining a new patient**	Creating profiles for Iranian and non‐Iranian patients involves registering personal information (name, gender, date of birth), contact details (country, province, city, address, phone number), and medical records (system record number, medical record number, registration date), as well as editing patient profiles.
3.	**Past medical history**	Record information such as: history of underlying disease, drug use, smoking, history and duration of hospitalization, reason for upper limb disability, affected hand (left, right, or both), presence and type of deformity or mass in the upper limb, history of movement disorders, amputation, hemiparesis, hemiplegia, rotator cuff syndrome, carpal tunnel syndrome, upper limb spasticity, degenerative arthritis/decreased joint range of motion, birth paralysis or brachial plexus injury, arterial injury in the upper limb, list of medications, and type of dominant hand (left, right, or both).
4.	**Patient's current history**	Recording information such as: the presence of wounds in the upper limb, musculoskeletal pains, numbness, tingling, muscle stiffness, inflammation and swelling of the joints, the presence of tendon injuries, tremors in the upper limb, contraction in the joints of the upper limb, range disabled limb movements, infection in the upper limbs, soft tissue necrosis, and weakness in the upper limbs
5.	**Levels of individual independence of the patient in performing daily activities**	Recording information such as: types of daily activities, types of daily activities dependent on others (for example, bathing, eating, dressing etc.), types of social participation (with family, friends, other people)
6.	**Patient's mental health problems**	Recording information such as: history of depression after upper limb disability, history of anxiety and distress after upper limb disability, need for social support and referral to another specialized treatment center, and need for social support at work and referral to another specialized treatment center
7.	**Treatment processes**	Including: determining the need for Bronstrom, prescribing the patient's exercises for rehabilitation, determining the stage and dose of repeating rehabilitation exercises, prescribing upper limb orthoses, prescribing medication, prescribing botulinum toxin, the need to measure muscle function with EMG, and prescribing other laboratory tests
8.	**Dashboard**	Providing statistical information such as the number of registered patients (temporary, confirmed), the number of current histories registered (temporary, confirmed, overall) and the number of follow‐ups (temporary, confirmed, overall) in the form of charts and statistical reports
9.	**Information registration menu**	Including sub‐menus for registering new patient's basic information, patient follow‐up, patient search (based on national ID, identification number, records number, name, surname and date of registration) and follow‐up list of patients
10.	**Reports menu**	Reporting of information recorded in the software in Excel and PDF formats
11.	**Follow‐ups list**	Showing follow‐ups of patients for the next 7 days, as well as patients who have not visited
12.	**Patient search menu**	Searching patients based on national ID, medical record number, first name, last name, and date of registration

It should be noted that after the registration of each patient in the software, if they have another visit, information on the type of visit (outpatient and inpatient), current medical history of the patient, the levels of individual independence of the patient in performing daily activities, patient's mental health problems, and treatment processes are recorded for each follow‐up

The Table 2 shows the demographic data of the participants in the usability evaluation of the registry software of upper limb disabilities. The frequency of women was more than men (seven women). Most of the participants were in the age group of 33–42 (61.53%). Moreover, most of the evaluators have a Ph.D. degree (76.9%).

**TABLE 2:** Participants’ demographics.

Variables	Frequency (percent)
**Sex**	
Male	6 (53.8)
Female	7 (46.2)
**Age**	
25‐34	2 (15.38)
35–44	8 (61.53)
45–54	3 (23.7)
**Education level**	
Master	3 (23.7)
PhD	10 (76.9)
**Field of study**	
Medical informatics	10 (76.9)
Health information management	3 (23.7)

As shown in Table 3, the mean obtained for all dimensions of the usability, i.e. “overall reaction to the software ”, “screen”, “terminology and information used in the software ”, “learning” and “ software capabilities” was achieved in the range of 6.1 to 9. Therefore, it can be said that the participants evaluated the usability of the registry software in all dimensions at the “good” level.

**TABLE 3:** Results of evaluation different aspects of the registry.

Evaluation aspects	Questions about each aspect	Mean (SD)
Overall reaction to the software	General use of the software	7.46(1.50)
	Ease of use of the software	7.08(1.84)
	How the user feels about using the software	7.15(1.76)
	General design of the software	7.00(2.27)
	Consistent use of the software	6.62(1.96)
	The settings feature of the software	6.69(2.07)
Screen	Reading characters on the screen	8.45(0.77)
	Using clear statement to simplify tasks	7.46(2.47)
	Organization of information	7.37(1.66)
	Sequence of screens	7.15(1.28)
Terminology and information used in the software	Use of terms throughout the software	7.77(1.42)
	Task‐related terminology	7.46(1.39)
	Position of messages on the screen	7.46(1.39)
	Prompts for input	7.69(1.37)
	software messages to complete user's tasks	7.69(1.45)
	Error messages	7.85(1.28)
Learning	Learning to operate the software	7.54(1.98)
	Exploring new features by trial and error	7.15(2.34)
	Remembering names and use of commands	7.46(1.98)
	Straightforward task performance	7.31(2.01)
	Help messages on the screen	7.31(2.53)
	Supplemental reference materials	6.46(1.96)
Software capabilities	Software speed	7.77(1.53)
	Software reliability	7.77(1.23)
	Number of software specifications	7.08 (1.44)
	Correcting user's mistakes when inputting data	6.92(2.01)
	Designed for all levels of users	7.08(2.21)

The highest and lowest mean of usability was “Reading characters on the screen” and “Registry software usage guide” respectively.

In the open question section, two evaluators mentioned the problem of not having an independent help icon on each page. It should be noted that this problem was fixed after the detection.

## 4 DISCUSSION

In the present study, registry software for upper limb disabilities was designed and then evaluated. In the following, both the design and development of registry software and its usability evaluation are discussed.

### 4.1 Design and develop of registry software of upper limb disabilities

Our registry software has capabilities for recording information such as the past medical history, the current medical history of the patient, the levels of individual independence of the patient in performing daily activities, information related to the patient's mental health problems, and the treatment processes performed for the patient. The designed software also has capabilities to create a profile for each user, define a new patient, search for individual and group patients, and individual or group reporting in the form of graphic diagrams, Excel and PDF files.

Various studies [23, 24, 25, 26] have been done to design and evaluate registries. Some of these designed registries had capabilities to record patients' clinical‐demographic information, advanced search, contact the site manager, provide news about the disease (prevention, self‐care, treatment information etc.) and provide daily statistics [24]. Creating profiles for data recording users, defining a new patient (recording demographic information, family status and history, treatment and complications form), searching, reporting, changing the condition of the record from pending to valid or vice versa, or removing either the entire record or individual follow‐ups of a patient were also among the features of the diabetes registry designed by Subhani and Al‐Rubeaan [27]. Showing the frequency distribution of variables such as gender, type of disease, marital status, disease complications, and type of treatment in the form of tables and graphs were among the other capabilities of the registry software designed by Subhani and Al‐Rubeaan [27]. Garavand et al.’s study [23] also showed that a registry software should be able to prepare different reports (reports in formats such as PDF and Excel files) to send to organizations and calculate important disease indicators. This registry software also has capabilities to record demographic information, hospitalization information, insurance information, medical history, medications and laboratory test results, risk factors, physician examination, data related to noninvasive procedures or invasive and surgical procedures, angiography findings, patient's state during discharge, and patient follow‐up [23]. Another study [28] has shown that the electronic registration of patient information in a registry software has greatly reduced the need to record information on paper and the number of repeated calls. Also, it has enabled registry staff to use information collected during previous contacts with registrants and registration candidates, eliminating the need to “cold call” potential registrants or study participants [28]. Moreover, the comprehensive capabilities and features of a registry can lead to the production of quality data [29].

In other words, the features and capabilities of a registry software should be such that they fulfill the purpose of a registry to produce high‐quality data [30]. Arts et al. [31], stated that the value of a registry depends on the quality of its data. High‐quality data from a registry are used to assess and improve the quality of care, examine treatment outcomes, evaluate treatment methods, and gain insight into the most cost‐effective treatment approaches based on the principles of value‐based health care [32]. Therefore, considering the capabilities and features that lead to the guarantee of data quality and validity is of great importance in the design of a registry software. Some studies have shown that many reasons can cause the production of poor quality or incomplete data, the most important of them are improper design of a registry software based on unnecessary characteristics, errors caused by wrong programming process of the system, manual entry of data into the software and failure to adhere to data definitions when filling out the forms [31]. To overcome these challenges, according to the opinion of experts the necessary capabilities and features for registry design should be identified, programming problems should be identified and corrected by the programming team, and the manual processes of entering data into the registry should be converted into automatic selection menus. Moreover, with the necessary training for the system users, as much as possible, they were introduced to the principles of documentation and the benefits of producing high‐quality and accurate data, and finally, the overall quality of the registry is always evaluated.

### 4.2 Usability evaluation of registry software of upper limb disabilities

In the present study, the mean obtained in different usability evaluation dimensions was in the range of 6.1 to 9. Therefore, the evaluators evaluated the various dimensions of the designed software at the “good” level. Usability evaluation of registries is also done in various studies [33, 34, 35]. The usability of registry software is crucial for its success and the quality of data it produces. High usability ensures that users can operate the system efficiently and accurately, which in turn supports high‐quality data collection. Poor usability can lead to increased user errors, reduced efficiency, and ultimately a negative impact on healthcare services. This has been supported by various studies, which highlight the importance of user‐friendly design and adequate training for users [36, 37]. Arab Kermani et al. [33], also evaluated the vitiligo  registry from the aspect of usability. This registry showed an acceptable level of usability according to the opinion of users by scoring 77.79 points. Most of the users (mean = 4.41) believed that this registry has the necessary integrity at different levels of performance, and its use has increased their confidence. Moreover, most users (mean = 4.24) had the tendency to use the registry frequently. Although users believed that the system was not complicated (mean = 1.59) and easy to use (mean = 1.35), they needed training by a technician to operate the system (mean = 2.18). [33].

According to these findings, Arab Kermani et al. [33], stated that the high usability of the software from the users' point of view indicates the success of the system and the strong correlation between the system and the users. On the other hand, poor usability of the system and unwillingness to use it continuously can cause poor return on investment and decrease productivity. Other studies [38, 39] have reported that the usability problems of an information system can increase user error and reduce the effectiveness and trust in the system, and ultimately have a negative impact on the quality of health care services provided. Failure to pay attention to these problems can affect users' interaction with the designed systems and in the long run lead to fatigue and abandonment of the system by users [33]. By evaluating the usability of the dementia registry through think‐aloud, Reichold et al. [35], also identified six categories of problems related to language, feedback, perceived suggested personality, design inconsistencies, orientation, and knowledge error. In order to overcome these problems, they needed to train users, provide a document with translation and explanations, complete translation of the texts used in the registry, provide a test system for assistance based on user expertise, provide a test system for optimizing the user interface, provide the type of keyboard based on field type (e.g. numeric keypad) and provide instructions for completing forms [35]. According to the study of Arab Kermani et al. [33], and Reichold et al. [35], it can be stated that one of the ways to improve usability, in addition to the simple and attractive design of systems, is to train people by a technician and provide a document to guide users to use the system easily.

In other words, ensuring usability of a system is one of the basic requirements for providing high‐quality services and meeting people's needs. Usable system can help users to do things quickly and easily. Furthermore, usability is directly related to clinical productivity, user fatigue, error rate, as well as user satisfaction. Some studies [33, 36, 40] showed that the poor usability of registries can affect the performance of data recording and lead to fatigue, decrease productivity and increase errors in data recording, and ultimately lead to a negative impact on the quality of health care services provided [33]. Moreover, poor usability can reduce the efficiency and effectiveness of systems, increase costs, reduce the compatibility of interactive systems with users and their tasks, and increase the negative outcomes of using a system [41].

Therefore, our study underscores the importance of focusing on usability and data quality in the design and evaluation of medical registry software. By addressing these aspects, we can ensure that the software effectively supports healthcare professionals and contributes to the improvement of patient care and treatment outcomes.

## 5 LIMITATIONS OF THE STUDY

The limitations of this study include the limited number of participants in the evaluation process and the short duration of the evaluation. It is suggested to consider the limitation of sample size and duration of evaluation in other studies.

## 6 CONCLUSION

Registry software for upper limb disabilities was designed and evaluated. The designed software can record and store patient data, determine the incidence and prevalence of disability in different parts of the upper limb, create a basis for producing clinical‐rehabilitation evidence, and evaluate preventive and therapeutic services provided to patients. Furthermore, this registry can lead to the production of quality data, provide a suitable platform for conducting various health‐clinical researches, establish communication between research groups and provide reliable evidence for policymaking and health planning at the national level.

The present study can also provide background knowledge for the design and development of other registers related to other body disabilities such as lower limb disabilities. Moreover, planners, policymakers and designers of disease registries can use this registry software as a model for designing and implementing other registries.

## AUTHOR CONTRIBUTIONS

**Khadijeh Moulaei**: Conceptualization; data curation; funding acquisition; investigation; project administration; resources; software; writing—original draft; writing—review and editing. **Abbas Sheikhtaheri**: Conceptualization; investigation; resources; supervision; writing—original draft; writing—review and editing. **AliAkbar Haghdoost**: Conceptualization; investigation; supervision; writing—review and editing. **Mansour Shahabi Nezhad**: Conceptualization; data curation; supervision; writing—review and editing. **Kambiz Bahaadinbeigy**: Conceptualization; data curation; funding acquisition; investigation; project administration; supervision; writing—review and editing.

## CONFLICT OF INTEREST STATEMENT

The authors declare no conflicts of interest.

## Data Availability

The data that support the findings of this study are available from the corresponding author upon reasonable request.
